# Potential for the International Spread of Middle East Respiratory Syndrome in Association with Mass Gatherings in Saudi Arabia

**DOI:** 10.1371/currents.outbreaks.a7b70897ac2fa4f79b59f90d24c860b8

**Published:** 2013-07-17

**Authors:** Kamran Khan, Jennifer Sears, Vivian Wei Hu, John S Brownstein, Simon Hay, David Kossowsky, Rose Eckhardt, Tina Chim, Isha Berry, Isaac Bogoch, Martin Cetron

**Affiliations:** Department of Medicine, Division of Infectious Diseases, University of Toronto, Toronto, Canada; Keenan Research Centre, Li Ka Shing Knowledge Institute, St. Michael's Hospital, Toronto, Canada; Keenan Research Centre, Li Ka Shing Knowledge Institute, St. Michael's Hospital, Toronto, Canada; Keenan Research Centre, Li Ka Shing Knowledge Institute, St. Michael's Hospital, Toronto, Canada; Children’s Hospital Boston, Harvard Medical School, Boston, USA; Spatial Ecology and Epidemiology Group, Department of Zoology, University of Oxford, Oxford, UK; Keenan Research Centre, Li Ka Shing Knowledge Institute, St. Michael's Hospital, Toronto, Canada; Keenan Research Centre, Li Ka Shing Knowledge Institute, St. Michael's Hospital, Toronto, Canada; Keenan Research Centre, Li Ka Shing Knowledge Institute, St. Michael's Hospital, Toronto, Canada; Keenan Research Centre, Li Ka Shing Knowledge Institute, St. Michael's Hospital, Toronto, Canada; Department of Medicine, Division of Infectious Diseases, University of Toronto, Toronto, Canada; University Health Network, Divisions of Internal Medicine and Infectious Diseases, Toronto, Canada; Division of Global Migration and Quarantine, Centers for Disease Control and Prevention, Atlanta, USA; Departments of Medicine and Epidemiology, Emory University School of Medicine and Rollins School of Public Health, Atlanta, USA

## Abstract

Background: A novel coronavirus (MERS-CoV) causing severe, life-threatening respiratory disease has emerged in the Middle East at a time when two international mass gatherings in Saudi Arabia are imminent. While MERS-CoV has already spread to and within other countries, these mass gatherings could further amplify and/or accelerate its international dissemination, especially since the origins and geographic source of the virus remain poorly understood. Methods: We analyzed 2012 worldwide flight itinerary data and historic Hajj pilgrim data to predict population movements out of Saudi Arabia and the broader Middle East to help cities and countries assess their potential for MERS-CoV importation. We compared the magnitude of travel to countries with their World Bank economic status and per capita healthcare expenditures as surrogate markers of their capacity for timely detection of imported MERS-CoV and their ability to mount an effective public health response. Results: 16.8 million travelers flew on commercial flights out of Saudi Arabia, Jordan, Qatar, and the United Arab Emirates between June and November 2012, of which 51.6% were destined for India (16.3%), Egypt (10.4%), Pakistan (7.8%), the United Kingdom (4.3%), Kuwait (3.6%), Bangladesh (3.1%), Iran (3.1%) and Bahrain (2.9%). Among the 1.74 million foreign pilgrims who performed the Hajj last year, an estimated 65.1% originated from low and lower-middle income countries. Conclusion: MERS-CoV is an emerging pathogen with pandemic potential with its apparent epicenter in Saudi Arabia, where millions of pilgrims will imminently congregate for two international mass gatherings. Understanding global population movements out of the Middle East through the end of this year's Hajj could help direct anticipatory MERS-CoV surveillance and public health preparedness to mitigate its potential global health and economic impacts.

## Introduction

As of July 12th 2013, 81 cases of the Middle East Respiratory Syndrome (MERS) have been confirmed worldwide, with Saudi Arabia reporting approximately 80% of cases. 1 ^,^ 2 Among confirmed infections, MERS has a mortality rate exceeding 50%, has a spectrum of illness that includes asymptomatic infection, mild illness and life-threatening severe disease, 3 ^,^ 4 ^,^ 5 ^,^ 6 appears to cause more severe disease in individuals with underlying medical comorbidities, 3 ^,^ 4 ^,^ 6 ^,^ 7 ^,^ 8 has demonstrated its potential for community and hospital based human-to-human transmission, 3 ^,^ 4 ^,^ 9 ^,^ 10 and has already dispersed to the United Kingdom, France, Germany, Tunisia and Italy. While the MERS coronavirus (MERS-CoV) is genetically similar to coronaviruses found in bats 11 ^,^ 12 and is suspected to be of animal origin, no animal source has yet been identified.

Every year, millions of domestic and foreign Muslim pilgrims congregate in Saudi Arabia to perform Umrah and Hajj. Umrah, which is a “lesser” pilgrimage, may be performed at any time of year but is considered particularly auspicious during the month of Ramadan (July 9^th^ to August 7^th^ in 2013), when it will draw an estimated one million pilgrims. By comparison, the Hajj is a five-day pilgrimage that is required of all physically and financially able Muslims at least once in their lifetime. This year (October 13^th^ to 18^th^), the Hajj is expected to draw over three million pilgrims from within Saudi Arabia and around the world. In 2012, 55% of Hajj pilgrims (1.74 million) were of foreign origin. 13

In light of the potential for these imminent mass gatherings to amplify MERS-CoV cases and contribute to its international spread,^14^^,^ 15 we describe worldwide air travel patterns out of the Middle East between Ramadan and the month following the Hajj (since foreign pilgrims are required to leave Saudi Arabia within one month of the Hajj) as a means to anticipate the most likely pathways of spread. Knowledge of how the international community is connected to the Middle East through air travel during this time period is not well understood, but could help cities and countries worldwide optimally distribute their finite resources for enhanced MERS-CoV surveillance and public health preparedness. Furthermore, it could help the international community identify countries that have strong travel connections to Saudi Arabia and the Middle East but which have limited capacity to detect MERS-CoV in a timely manner, and if necessary, mobilize an effective public health response to imported cases including the implementation of rigorous infection control practices. Adopting a proactive and anticipatory approach to the international spread of MERS-CoV may help to mitigate its global health and economic consequences.

## Methods

Using global data from the International Air Transport Association (IATA), we analyzed the anonymized flight itineraries of all travelers who embarked on commercial flights, including scheduled charters, from any domestic or international airport within Saudi Arabia, Jordan, Qatar and the United Arab Emirates (UAE) and who disembarked at an international destination between the months of June and November 2012 (i.e. encompassing one month before Ramadan and one month after the Hajj). IATA captures 93% of the world’s commercial air traffic data and uses market intelligence to produce estimates for the remainder. Each flight itinerary encompassed data on the city where each traveler initiated their trip along with all connecting flights en route to their final destination. For all travelers initiating trips within the aforementioned four countries, not including persons transiting through these countries from other international points of origin, we calculated the total number of travelers with final destinations at each international airport worldwide. Saudi Arabia, Jordan, Qatar and UAE were selected as the points of origin for travelers in this descriptive statistical analysis because to date, all confirmed MERS-CoV cases in international travelers have been linked back to these four countries. We then compared these global travel patterns with those observed during the previous five years (i.e. June to November for each year from 2007 to 2011) to determine if there was a consistent seasonal pattern. As a large proportion of foreign Hajj pilgrims travel to and from Saudi Arabia via unscheduled charter flights, which are not captured in the aforementioned analysis, we concurrently modeled the expected national origins of Hajj pilgrims in 2013. To do this, we multiplied the total number of foreign Hajj pilgrims in 2012 13 by the proportions of pilgrims originating from individual countries based on Muslim population estimates from the Saudi Ministry of Hajj and medical literature, 16 ^,^ 17 and assumed a comparable number of pilgrims in 2013 relative to 2012. We then compared these values with the World Bank economic classifications of countries and estimates of national healthcare expenditures per capita 18 as simplified, albeit imperfect surrogate markers of public health capacity (i.e. their ability to detect imported MERS-CoV in a timely manner and mobilize an effective public health response to such cases).

## Results

16.8 million travelers on commercial flights departed Saudi Arabia, Jordan, Qatar and UAE for an international destination between June and November 2012. 7.5% had final destinations in countries that were low income, 47.4% lower-middle income, 17.3% upper-middle income and 27.8% high income. 51.6% had final destinations in just eight countries: India (16.3%), Egypt (10.4%), Pakistan (7.8%), the United Kingdom (4.3%), Kuwait (3.6%), Bangladesh (3.1%), Iran (3.1%) and Bahrain (2.9%; see Table). Individual cities with the highest travel volumes include Cairo, Kuwait City, London, Bahrain, Beirut, Mumbai, Dhaka, Karachi, Manila, Kozhikode, Istanbul and Jakarta, each of which received more than 350,000 commercial air travelers from MERS-CoV source countries between June and November 2012. Furthermore, an estimated 8.7% of foreign Hajj pilgrims in 2012 originated from countries that were low income, 56.4% lower-middle income, 27.3% upper-middle income, and 7.6% high income. 60.7% of foreign pilgrims originated from just eight countries – Indonesia (12.4%), India (10.1%), Pakistan (9.9%), Turkey (7.8%), Iran (6.5%), Nigeria (5.7%), Egypt (5.5%) and Bangladesh (2.9%). A bubble plot depicting the volume of international travelers departing Saudi Arabia, Jordan, Qatar and UAE from June to November 2012, the estimated number of foreign pilgrims performing the Hajj in 2012 and estimated healthcare expenditures per capita in 2011 is shown in Figure 1.

**Country-Level Destinations of Air Travelers Departing MERS-CoV Source Countries*, Origins of Hajj Pilgrims†, and Healthcare Expenditures per Capita‡:**
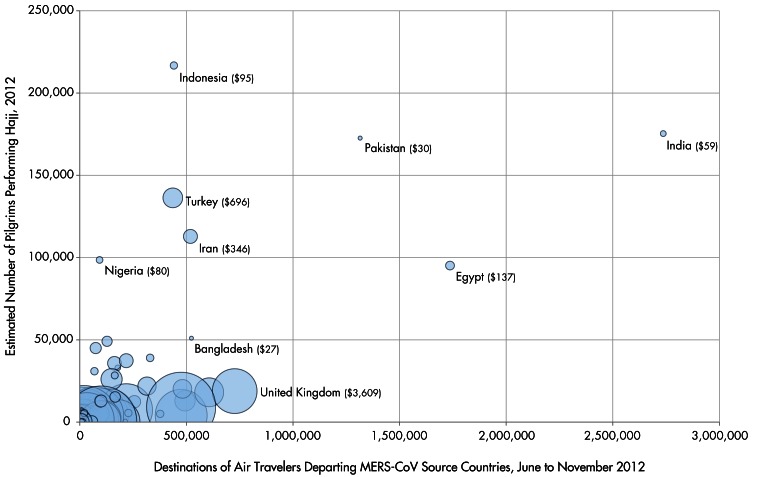
* Final Destinations of Air Travelers Departing Saudi Arabia, Jordan, Qatar and the United Arab Emirates via Commercial Flights between June and November 2012† Estimated for 2012 ‡ Sizes of the circles are proportionate with healthcare expenditures per capita as estimated by the World Bank, 2011

A world map depicting the city level destinations of travelers and the national origins of foreign Hajj pilgrims is shown in Figure 2, demonstrating that while no region of the world is isolated, Central and South America see the fewest number of travelers.

**City-Level Destinations of Air Travelers Departing MERS-CoV Source Countries* and Origins of Hajj Pillgrims†:**
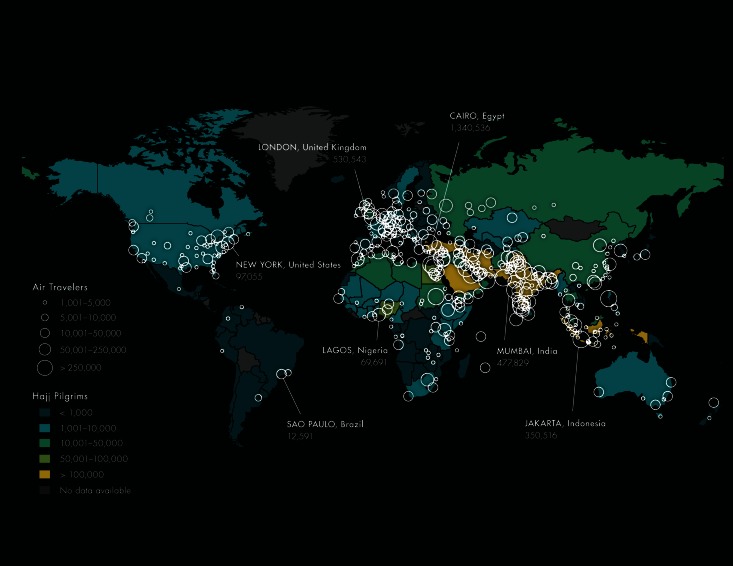
* Final Destinations of Air Travelers Departing Saudi Arabia, Jordan, Qatar and the United Arab Emirates via Commercial Flights between June and November 2012 † Estimated for 2012

**Table: Country-Level Destinations of Air Travelers Departing MERS-CoV Source Countries*, Origins of Hajj Pilgrims†, and Healthcare Expenditures per Capita‡ by World Bank Economic Status§:** * Final Destinations of Air Travelers Departing Saudi Arabia, Jordan, Qatar and the United Arab Emirates via Commercial Flights between June and November 2012. Restricted to countries with at least 10,000 travelers or 1000 pilgrims. † Estimated assuming the same number of foreign pilgrims in 2013 compared with 2012 ‡ World Bank 2011 § World Bank 2013 ** Sudan pilgrim values include those from South Sudan †† China traveler and pilgrim values include Hong Kong and Macau

Country	Final Destinations of Air Travelers Departing the Middle East	Estimated Hajj Pilgrims	Healthcare Expenditures per Capita (US$)
**Low Income Countries**			
Bangladesh	523,643	51,021	$27
Nepal	213,343	509	$33
Afghanistan	178,477	33,011	$56
Ethiopia	126,103	4,009	$17
Kenya	90,044	2,480	$36
Tanzania	23,682	2,000	$37
Uganda	18,645	877	$42
Somalia	14,731	6,540	No Data
Kyrgyzstan	9,032	No Data	$71
Tajikistan	8,465	6,965	$54
Mauritania	5,171	3,227	$58
Myanmar	4,641	3,382	$23
Niger	3,594	8,329	$20
Chad	2,870	5,314	$35
Mali	2,180	6,604	$45
Togo	1,530	1,397	$45
Burkina Faso	982	2,378	$37
Guinea	604	5,861	$30
Benin	452	3,718	$37
Gambia	375	1,496	$27
**Lower-Middle Income Countries**			
India	2,736,782	175,334	$59
Egypt	1,736,579	95,138	$137
Pakistan	1,315,089	172,610	$30
Indonesia	441,557	216,716	$95
Philippines	377,251	4,992	$97
Sudan	329,927	39,022**	$104
Sri Lanka	228,051	5,492	$97
Yemen	164,040	28,353	$88
Iraq	162,639	35,748	$332
Morocco	127,767	49,062	$186
Nigeria	93,017	98,559	$80
Syria	68,832	30,921	$101
Ukraine	56,163	177	$263
Uzbekistan	20,494	5,625	$88
Djibouti	15,334	1,369	$105
Viet Nam	12,512	No Data	$95
Ghana	11,939	2,580	$75
Armenia	11,775	No Data	$142
Senegal	11,348	8,139	$67
Georgia	10,802	5,587	$328
South Sudan	5,441	No Data**	$32
Cote D'Ivoire	4,148	3,786	$79
Cameroon	3,760	3,570	$68
**Upper-Middle Income Countries**			
Iran	518,929	112,843	$346
Lebanon	481,546	20,183	$622
Turkey	436,218	136,301	$696
China	312,056††	12,370††	$278
Malaysia	217,993	37,317	$346
Thailand	165,717	15,259	$202
Russia	149,026	26,056	$807
Tunisia	98,383	12,819	$267
South Africa	91,573	5,061	$689
Algeria	74,848	45,015	$225
Libya	70,001	10,983	$398
Maldives	51,639	1,133	$545
Kazakhstan	49,633	2,646	$455
Azerbaijan	39,153	5,321	$357
Seychelles	37,982	No Data	$439
Mauritius	22,292	1,897	$510
Brazil	21,899	89	$1,121
Angola	21,169	25	$186
Romania	20,838	171	$500
Turkmenistan	19,440	885	$129
Serbia	14,542	1,563	$622
Macedonia	4,641	2,267	$334
Bosnia and Herzegovina	3,949	2,545	$493
**High Income Countries**			
United Kingdom	726,506	18,827	$3,609
Kuwait	607,090	18,085	$1,500
Bahrain	493,624	12,914	$740
Germany	475,445	4,081	$4,875
United States of America	475,355	9,272	$8,608
Oman	315,346	21,845	$598
France	218,272	7,322	$4,952
Italy	179,866	313	$3,436
Australia	129,372	2,945	$5,939
Singapore	110,106	2,350	$2,286
Switzerland	100,677	338	$9,121
Spain	97,083	582	$3,027
Canada	96,950	3,548	$5,630
Netherlands	70,655	3,972	$5,995
Austria	68,239	1,047	$5,280
South Korea	63,055	7	$1,616
Japan	56,580	28	$3,958
Denmark	35,882	810	$6,648
Greece	34,594	648	$2,864
Sweden	34,019	1,334	$5,331
Ireland	31,931	122	$4,542
Belgium	22,887	1,251	$4,962
Czech Republic	22,382	39	$1,507
Cyprus	21,903	20	$2,123
Norway	17,030	801	$8,987
Portugal	15,307	89	$2,311
Poland	14,096	46	$899
New Zealand	13,571	283	$3,666
Finland	10,137	97	$4,325
Brunei Darussalam	2,976	1,149	$993

## Discussion

At the time of writing, imported MERS-CoV has been confirmed in Germany, the United Kingdom, France, Italy, and Tunisia, with the latter four countries reporting domestic transmission. Given that the destinations of international travelers departing Saudi Arabia, Jordan, Qatar and UAE have shown a highly consistent seasonal pattern from 2007-2012, our findings suggest that India, Egypt, Pakistan, the United Kingdom, Kuwait, Bangladesh, Iran and Bahrain will likely account for more than half of the world’s air traffic out of the aforementioned Middle Eastern countries from June to November this year, and hence have significant potential for MERS-CoV introduction via commercial flights. Furthermore, with millions of foreign pilgrims set to congregate in Mecca and Medina between Ramadan and Hajj, pilgrims could acquire and subsequently return to their home countries with MERS-CoV, either through direct exposure to the as of yet unidentified source or through contact with domestic pilgrims who may be infected. Our findings also indicate that two-thirds of all Hajj pilgrims will be returning to low or lower-middle income countries where medical and public health capacity will be limited, and presumably where the risk of domestic transmission of imported MERS-CoV will be elevated.

Public health interventions to prevent or attenuate the international spread of an emerging infectious disease and its subsequent domestic consequences may be directed at three frontiers: the source area(s), travelers departing the source area(s), and geographies worldwide receiving travelers from the source area(s). In the case of MERS-CoV, rigorous efforts in Saudi Arabia and the Middle East are ongoing to identify the presumed animal origins of this novel human coronavirus. While there is presently no evidence to indicate that animal livestock are a direct or intermediate source of MERS-CoV, reasonably excluding this possibility will be important since millions of animals, including goats, sheep, cattle and camels will be sacrificed during Ramadan and at the end of Hajj (i.e. Eid-al Adha or Festival of Sacrifice). Products of these animal sacrifices will then be consumed by pilgrims and distributed to the poor.

Many Middle Eastern countries are home to large foreign-born populations including migrant workers (e.g. an estimated 28.7% of Saudi Arabia’s population is comprised of foreign nationals), 19 which may be at risk for MERS-CoV acquisition and subsequent spread to their home countries. These populations may be at heightened risk due to inadequate healthcare access in their host country (e.g. lack of health insurance, language barriers) and may be “undercounted” from traditional government disease surveillance systems.

The merits of traveler health screening as an intervention to reduce the risks, either perceived or real, of the international spread of infectious disease is often debated.^20^^,^ 21 ^,^ 22 ^,^ 23 ^,^ 24 A recent study evaluating the evidence for traveler health screening worldwide during the 2009 H1N1 influenza pandemic indicated that while the effectiveness of screening can vary depending upon a number of factors related and unrelated to the pathogen of interest, the efficiency of screening can be increased significantly by focusing on a small number of pivotal airports. 22 Furthermore, this study indicated that for a pathogen such as MERS-CoV with a median incubation period of 5 days and an upper limit of 14 days, 2 ^,^ 10 effective exit screening of travelers departing known source areas in the Middle East would preclude the need for entry screening elsewhere in the world. 22 This strategy would align with the purpose of the International Health Regulations to prevent the international spread of infectious disease while minimizing “interference with international traffic and trade”. 25

Finally, countries receiving pilgrims and other travelers from known MERS-CoV source areas should mobilize their infectious disease surveillance and public health resources in ways that are commensurate with their predicted risks of MERS-CoV importation. Educating and preparing front line healthcare providers to consider the diagnosis of MERS-CoV is critical, since the timely implementation of effective infection control practices within communities and healthcare institutions is contingent upon providers’ clinical index of suspicion. In the SARS epidemic, delays in the implementation of appropriate infection control measures stemming from delays in considering the diagnosis, as well as infection control breaches among suspected or confirmed SARS cases remains a painful reminder of the critical role of infection control. However, it is recognized how difficult this may be for resource poor countries that have strong travel connections to Saudi Arabia and the broader Middle East, but lack sufficient medical and public health resources to detect MERS-CoV in a timely fashion, and if necessary, mobilize an effective public health response.

Since MERS-CoV appears to have a predilection for individuals with underlying medical comorbidities, 2 ^,^ 3 ^,^ 4 ^,^ 6 ^,^ 7 ^,^ 8 Umrah and Hajj pilgrims – of whom many suffer from chronic illnesses and poor health^26^^,^ 27 ^,^ 28 ^,^ 29 ^,^ 30 – may be particularly vulnerable to infection and/or severe outcomes from MERS-CoV. Timely detection of MERS-CoV may be further confounded by its clinical spectrum of illness, which ranges from asymptomatic infection to mild illness to severe disease. 3 ^,^ 6 ^,^ 8 ^,^ 9 Early in the course of illness, gastrointestinal symptoms may lead clinicians down alternate diagnostic pathways. 2 ^,^ 3 ^,^ 4 Among returning Hajj pilgrims, undifferentiated respiratory infections sometimes referred to as the “Hajj cough” are common^27^^,^ 29 ^,^ 30 ^,^ 31 ^,^ 32 and may complicate diagnosis and management at a time when influenza-like-illnesses are expected to increase across the northern hemisphere, where most of the world’s Muslims reside. Furthermore, there are significant practical challenges to rapidly establishing MERS-CoV diagnostic capacity at a global level, especially in high-risk, resource poor countries. Since pilgrims are required to leave Saudi Arabia within one month of completing their Hajj pilgrimage (i.e. which ends on October 18^th^, 2013) and since MERS-CoV has an estimated incubation period of up to two weeks, 2 enhanced international surveillance post-Hajj should continue through at least the end of November 2013.

Our study has a number of limitations. Foremost, we were unable to analyze data on unscheduled charter flights since there are no centralized sources of such data. Thus, we performed parallel and complementary analyses of commercial air travelers departing the Middle East for destinations at the city-level alongside estimates of Hajj pilgrims at the national-level. Furthermore, we did not account for the approximately 10% of foreign pilgrims that travel to Saudi Arabia by land or sea from neighbouring countries to perform the Hajj. 17 We were also limited by an imprecise understanding of the spatial extent of the animal and/or environmental source of MERS-CoV within the Middle East, which required us to consider all domestic and international airports in Saudi Arabia,Jordan,Qatar and UAE. We categorized countries by their World Bank economic status and per capita healthcare expenditures as crude surrogate markers of their ability to detect MERS-CoV in a timely manner, implement effective infection control precautions, and mobilize a vigorous response to imported cases. Recognizing that there are no established indicators for this type of diagnostic, medical, and public health capacity, we produced tabular information at a national level to enable the integration of other potential indicators of interest.

The four countries with confirmed cases in returning travelers, not including international transfers of medical care, (i.e. the United Kingdom, France, Italy and Tunisia) 2 account for an estimated 7.1% of the final destinations of all international travelers departing the MERS-CoV source countries since September 2012 (each of which are high or upper-middle income countries). By comparison,India,Pakistan and Bangladesh represent the final destinations of an estimated 27.7% of all international travelers over the same time period (each of which are low or lower-middle income countries), but have not reported cases of MERS-Co. Although not definitive, these findings could indicate the presence of epidemiological “blind spots” to MERS-CoV as a result of limited infectious disease diagnostic and surveillance capacity.

Ten years after the emergence of a novel coronavirus in China’s Guangdong province,^33^ a new virus has emerged with its epicenter in the Middle East, and with a mortality rate among confirmed cases exceeding 50%. While the ability of this virus to spread from person-to-person thus far appears somewhat limited,^34^ super-spreading events as observed during SARS 33 could significantly alter the global course of this epidemic. Compared with SARS, which began to spread internationally in the spring of 2003 to predominantly high income countries with strong travel links to China, MERS will coincide with two mass gatherings in Saudi Arabia that are expected to draw millions of travelers and Muslim pilgrims from predominantly resource poor countries with limited capacity to detect and respond to imported cases.

The emergence of MERS-CoV requires an internationally coordinated effort to mitigate its potential global health and economic consequences, with particular emphasis on supporting diagnostic and public health response capacity in vulnerable, resource-limited countries. Understanding the most probable pathways for international spread of MERS-CoV could help medical and public health providers worldwide operate in a far more anticipatory and less reactive manner than occurred during SARS.

## Competing Interests

I have read the journal's policy and have the following conflict: Kamran Khan owns intellectual property rights in BioDiaspora, a technology dedicated to understanding the role of global travel in the international spread of infectious diseases. The other authors do not have any conflicts of interest to declare.

## Authors' Contributions

Kamran Khan led the study from its initial design to the preparation of the first and last drafts of the manuscript. Jennifer Sears contributed to the design and analysis of the study, along with preparation of and edits to the final version of the manuscript. Wei Hu performed all statistical analyses of air traffic data and helped prepare the final version of the manuscript. John Brownstein contributed epidemiological data pertaining to MERS-CoV and made significant content contributions and edits to the final manuscript. Simon Hay made significant content contributions and edits to the final manuscript. David Kossowsky created data visualizations pertaining to global travel patterns and contributed to the final version of the manuscript. Rose Eckhardt researched epidemiological data pertaining to MERS-CoV and made contributions to the final version of the manuscript. Tina Chim created data visualizations pertaining to global travel patterns and contributed to the final version of the manuscript. Isha Berry researched Ramadan and Hajj data and pilgrim practices and made contributions to the final version of the manuscript. Isaac Bogoch made significant content contributions and edits to the final manuscript. Martin Cetron made significant content contributions to the initial draft of the manuscript and edits to the final draft of the manuscript.
